# Effects of early enteral and parenteral nutrition support combined with “Internet + nursing service” on quality of life and complications in patients with malignant obstructive jaundice after percutaneous hepatic puncture biliary drainage

**DOI:** 10.3389/fsurg.2025.1688387

**Published:** 2025-11-26

**Authors:** Liping Hou, Huibo Li, Dongyan Zhang

**Affiliations:** 1 Department of Interventional Vascular, Shanxi Fenyang Hospital, Fenyang, Shanxi, China; 2 Department of Imaging Catheter Room, Shanxi Fenyang Hospital, Fenyang, Shanxi, China

**Keywords:** early enteral nutrition, Internet, nursing, malignant obstructive jaundice, parenteral nutrition, percutaneous transhepatic choledochus drainage

## Abstract

**Aim:**

We explored the effects of early enteral and parenteral nutrition support combined with “Internet + nursing service” on quality of life and complications in patients with malignant obstructive jaundice after percutaneous transhepatic choledochus drainage (PTCD).

**Methods:**

The control group (CG) adopted routine nursing and total parenteral nutrition support. The experimental group (EG) adopted “Internet + nursing service” and early enteral + parenteral nutrition support.

**Results:**

Compared with the CG, the EG demonstrated shorter times of first exhaust/first defecation and reduced length of hospital stay. The incidence of complications was lower, and scores for physical, emotional, social, and material life were higher. Nursing satisfaction of patients was better, while self-rating anxiety scale and self-rating depression scale scores were lower. Aminotransferase, total bilirubin, and direct bilirubin levels were reduced, and improvements in CD8^+^, CD4^+^, and CD4^+^/CD8^+^ levels were more pronounced. By the 7th day of treatment, serum albumin and prealbumin levels were higher in the EG than those in the CG.

**Conclusion:**

Early enteral and parenteral nutrition support combined with “Internet + nursing service” can promote the quality of life, improve nutritional status and immune function, and reduce the complications in patients with malignant obstructive jaundice after PTCD.

## Introduction

Malignant obstructive jaundice is primarily characterized by hyperbilirubinemia, tissue and body fluid staining, and bile duct dilatation caused by direct or indirect biliary obstruction from malignant tumors (1). With advancements in clinical graded diagnosis and treatment services and evolving rehabilitation concepts, an increasing number of patients with malignant obstructive jaundice undergo percutaneous transhepatic choledochus drainage (PTCD) to reduce serum bilirubin levels, quickly relieve jaundice symptoms, improve liver function, and prolong survival (2). PTCD involves percutaneous puncture into the intrahepatic bile duct, followed by injection of a contrast agent to visualize intrahepatic bile duct development and biliary drainage (3). However, patients often require long-term indwelling tubes after PTCD, which greatly reduces the quality of life of patients and adversely affects their physical and mental health (4). Therefore, effective postoperative rehabilitation interventions are critically important.

The “Internet + nursing service” is a new continuous nursing model that integrates big data and mobile Internet technologies to provide patients with convenient, efficient, and personalized nursing services through online application and offline on-site service (5). This model has been widely studied and applied in some cities in China and has become an important part of the healthcare reform (6).

Patients with malignant tumors are at high risk of malnutrition, with a prevalence of 40%–80% (7). Therefore, postoperative nutritional support is a necessary problem in surgical treatment, especially for patients with digestive tract tumors, and further treatment after surgery is an important topic. Patients suffer from varying degrees of malnutrition in the perioperative period due to tumor consumption and the influence of the disease on diet, etc (8). Malnutrition seriously affects the prognosis of patients and their tolerance to surgical treatment, can lead to increased postoperative complications, and directly affects the quality of life of patients (9). Therefore, when implementing radical or palliative surgical treatment for such patients, reasonable nutritional support after surgery is of great significance to the recovery of patients (10). Studies have shown that the peristalsis, digestion, and absorption functions of the small intestine can usually be recovered within 12–24 h after surgery (11). At present, early postoperative enteral nutrition is increasingly accepted by the majority of clinicians (12).

In this study, we explored the effect of early enteral and parenteral nutrition support combined with “Internet + nursing service” on quality of life and complications in patients with malignant obstructive jaundice after PTCD.

## Methods

### Patients

One hundred patients with malignant obstructive jaundice who received PTCD treatment in our hospital from January 2022 to December 2023 were enrolled as research objects. They were randomly divided into a control group (CG) and an experimental group (EG), with 50 cases in each group. The randomization was performed using a computer-generated random number sequence. The allocation sequence was concealed by using sequentially numbered, opaque, sealed envelopes (SNOSE), which were opened only after the patient had provided written informed consent and was enrolled in the study.

The CG included 30 males and 20 females, with an average age of 54.31 ± 6.86 years, ranging from 41 to 70 years. The EG contained 29 males and 21 females, with an average age of 54.34 ± 5.85 years, ranging from 42 to 72 years. No difference was discovered in general data between the two groups (*P* > 0.05). All patients signed informed consent forms.

### Inclusion and exclusion criteria

Inclusion criteria: (1) patients diagnosed with malignant obstructive jaundice by digital subtraction angiography (DSA), MRI, CT, etc. in our department, presenting with symptoms such as varying degrees of skin pruritus, dark yellow urine, loss of appetite, gray stool, and yellowing of the sclera and upper skin; (2) patients who could actively cooperate with this study; (3) patients with no communication disorder, cognitive disorder, mental disorder, and movement disorder patients; and (4) patients with complete clinical data.

Exclusion criteria: (1) patients with hematological, visceral, infectious, and immunosystemic diseases; (2) patients with hypertension and diabetes; and (3) patients with coagulation dysfunction.

### Nursing interventions

The CG received routine nursing, and the specific contents were as follows. (1) Health education: Nurses informed family members of the relevant knowledge and matters needing attention. (2) Dietary guidance: Nurses informed patients and their families that the diet of patients should be light, eat less and eat more, and drink more water. (3) Psychological counseling: Nurses encouraged family members to strengthen communication with patients and relieve the tension of patients and their families. (4) Disease knowledge guidance: Nurses explained the causes of postoperative pain and measures to relieve pain for patients and their families.

The EG adopted “Internet + nursing service,” and the specific scheme was as follows. (1) Establishing an Internet platform: With the help of the Internet company, a specialized nursing platform was established with the hospital as the core, including the information, nursing, medical, education, management, and service departments. The service staff of the platform was composed of hospital volunteers and social volunteers with nursing certificates. The information department was responsible for collecting patients' clinical information, treatment information, and adverse events during treatment and establishing independent electronic files, which could be viewed online by doctors and nurses with permission. The nursing department was composed of nurses and head nurses with rich experience in clinical nursing. The medical department was made up of experienced doctors from all departments of the hospital. The education department was responsible for health education, which meant that the postoperative rehabilitation nursing methods and precautions were pushed through videos and documents on the platform every day, and topics were selected by submitting contributions from patients, and expert lectures were held once a week. The management department was responsible for developing effective nursing intervention programs according to patients' nursing needs and assigning nursing teams to patients according to their wishes. The service department was responsible for receiving the patient's booking form and filling out the patient's questionnaire on the outcome of this care.

(2) Nursing process: Patients filled in their basic information, including disease diagnosis, treatment methods, admission and discharge time, nursing needs, and needs for nursing staff, through the service department of the specialized nursing platform (accessed through WeChat mini programs or apps). After filling in the information, the patient checked whether it was correct and submitted the order after confirming it was correct. After seeing the order, the staff of the management department of the specialty nursing platform checked the identity information of the patient, discussed it after the verification, formulated reasonable and scientific nursing contents for the patient, and set up a nursing team suitable for the patient. After the nursing team accepted the task, the hospital arranged a special car to pick up the service point, the first time after arrival to the patient or family members to show identification materials, to obtain the trust of patients and family members. After the completion of nursing work, nurses recorded the work content on the platform, which was managed by the information department. Patients paid fees on the platform and filled out evaluation questionnaires. Within 1 week after the completion of the nursing work, the platform management staff conducted a phone or WeChat follow-up visit to the patient to find the patient's unresolved problems in time.

(3) Specific nursing content: A nursing team suitable for patients was set up, which was composed of one experienced surgeon, one physician, one psychologist, one dietitian, one head nurse, and two nurses. The specific nursing contents were as follows. (1) Nurses explained the causes of pain after PTCD surgery, the impact of pain on life, and various complications and precautions for patients and their families and strengthened the awareness of patients and their families about the surgery. (2) The nurse demonstrated the fixed placement of the drainage tube to the patients and their families, adopted the “high lift method” to ensure the patency of the drainage tube, and periodically squeezed the tube to prevent liquid reflux and catheter blockage. (3) For patients with unbearable pain, analgesic drugs and liver protection drugs were given according to the doctor's advice, and patients were guided to do skin management. (4) Nurses closely monitored the patient's blood pressure, body temperature, blood sugar, and other indicators, observed the color of the fluid in the drainage tube, and determined whether internal bleeding occurred. (5) Dietitians developed a three-meals-a-day nutritional recipe according to the patient's favorite foods and nutritional needs, and the diet was based on a light liquid diet. (6) Psychologists communicated with patients to relieve their anxiety and anxiety and to inform family members of the skills of communication with patients and guided family members to play relaxing and pleasant music when patients had pain or anxiety.

### Nutrition support methods

The CG adopted total parenteral nutrition support. Patients were injected into the body at a constant rate from 12 to 15 h on the first day after surgery and continued for more than 7 days until the semi-liquid diet was eaten orally and parenteral nutrition was discontinued. Parenteral nutrition preparation was powered by 20% fat emulsion and glucose, the ratio of fat to sugar was 4:6, and the calorie intake was 130 kJ/(kg·d). The nitrogen content was 0.2 g/(kg·d), the ratio of calorie (kcal) to nitrogen content (g) was 150:1, and the nitrogen source was supplemented by compound amino acid (18AA-Ⅳ). The specification was 250 mL: 8.70 g (total amino acid). At the same time, the nutritional preparations, such as electrolytes, complex vitamins, and various trace elements, were supplemented with 3 L bags to make an all-in-one nutrient solution.

The EG adopted early enteral + parenteral nutrition support. During the operation, the patient's jejunal nutrition tube was pushed by the operator to 25–30 cm below the Treitz ligament or the subanastomotic output loop jejunum, and the catheter was properly fixed in the nasal alar. After the operation, intravenous nutrition support (preparation method was the same as that of the CG group) was provided. Additionally, 250 mL of normal saline was slowly dripped into the liquid capsule jejunal nutrition tube from the second day after the operation, and enteral nutrition preparations were dripped into the liquid capsule jejunal nutrition tube through the medical infusion pump on the third day after the operation. The selection of nutrition solution was enteral nutrition suspension [TPF, product name: Nutrison Fibre, manufactured by Nuditia Pharmaceutical (Wuxi) Co., LTD.; specification, 500 kcal per 500 mL solution]. The initial infusion volume was 300–500 mL/day, and the infusion rate was 20–30 mL/h. According to the patient's tolerance, the amount of parenteral nutrition was gradually increased, the amount of parenteral nutrition was reduced, and the TPF was gradually transitioned to the full dose (1,500–2,000 mL/day) within 2–3 days, and the drop rate was 100–125 mL/h. For more than 7 days, enteral nutrition was stopped after oral intake of a semi-fluid diet. The daily requirement of nutrients, such as enteral nutrition, was insufficient, and the rest was supplied by parenteral nutrition.

Both groups were given equal nitrogen and equal caloric nutrient solution; 40% of the total calorimetric calories came from fat, 60% from carbohydrates, and electrolytes were provided according to demand.

### Observation indicators

1. The time of first exhaust, the time of first defecation, and the length of hospital stay were compared between the two groups.
2. Self-rating anxiety scale (SAS) and self-rating depression scale (SDS) were used to compare the mood changes of both groups (13). The higher the score, the more severe the anxiety and depression symptoms were.
3. The incidence of complications, including biliary leakage, shock, intraperitoneal hemorrhage, and infection, was compared between the two groups.
4. The alanine aminotransferase (ALT), total bilirubin (TBIL), and direct bilirubin (DBIL) levels of the two groups before and 7 days after surgery were measured by Siemens (ADVI-A2400) automatic biochemical analyzer.
5. Five milliliters of fasting venous blood were collected from patients, and the nutritional status indexes of patients, including prealbumin (PAB) and serum albumin (ALB), were measured by the Mindray automatic biochemical analyzer.
6. Five milliliters of fasting venous blood were collected before treatment and 2 weeks after treatment, and the immune function indexes of the patients, including CD8^+^, CD4^+^, and CD4^+^/CD8^+^, were measured by flow cytometry.
7. The patients’ physical, emotional, social, and material life during the period of intervention was assessed by referring to the GQOLI-74 (14), which was measured on a scale of 100. The higher the score, the better the quality of life was.
8. The self-designed nursing satisfaction questionnaire was used to compare the satisfaction of patients in the two groups with nursing work. The full score of the questionnaire was 100 points, with satisfaction being 85–100 points; basic satisfaction, 70–84 points; and less than 70 points, dissatisfied. Total satisfaction rate = satisfaction rate + basic satisfaction rate.

### Statistical analysis

SPSS 24.0 statistical software was adopted for data analysis. Measurement data were expressed as (*x* ± *s*), and a *t*-test was adopted for comparison. Count data were expressed as (*n*, %), and the *χ*^2^ test was used for comparison. *P* < 0.05 meant statistical significance.

## Results

### Postoperative recovery between the two groups

**Figure 1:**
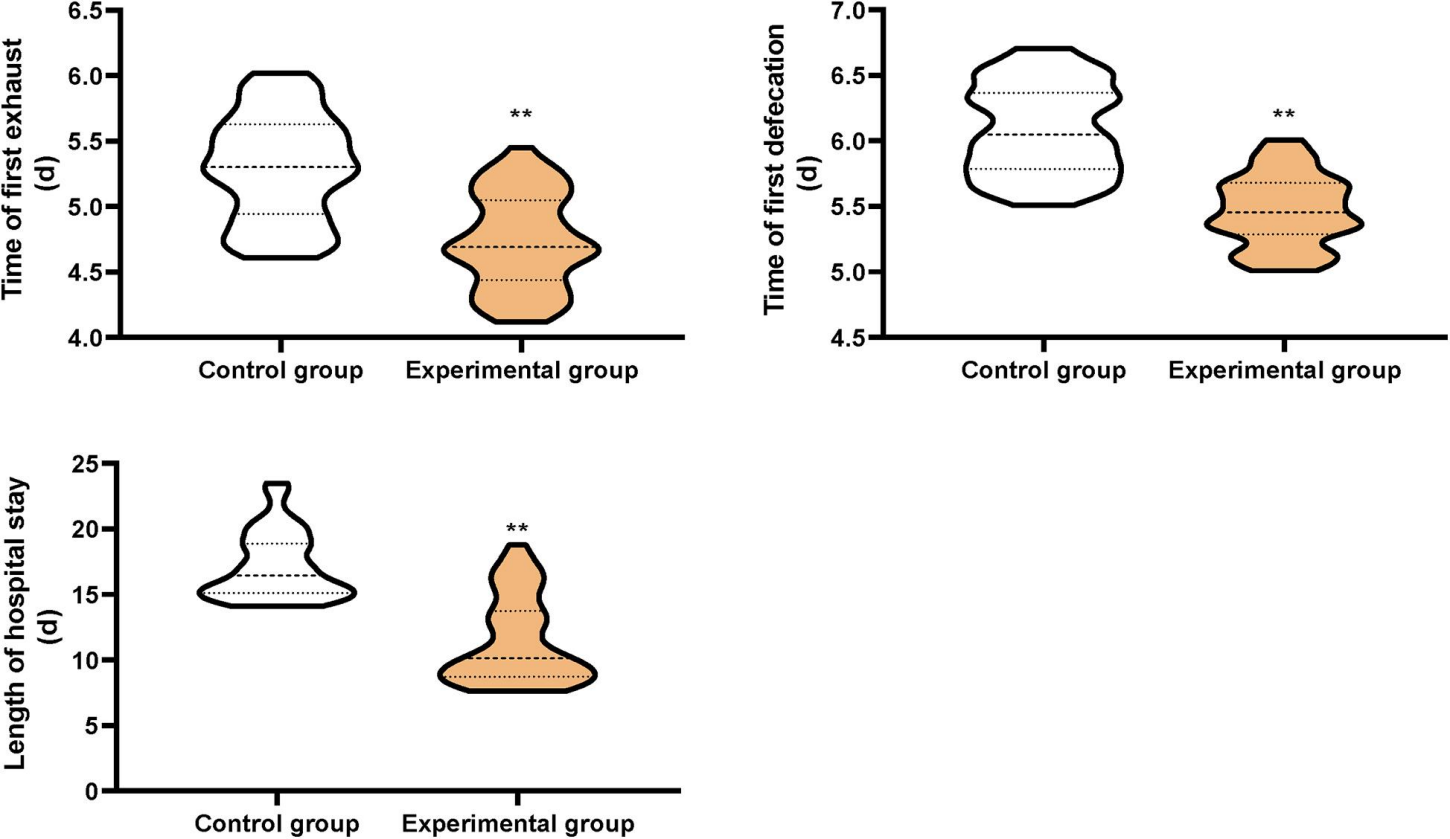
Postoperative recovery between the two groups. **P < 0.01.

Compared with the CG, the time of first exhaust, the time of first defecation, and the length of hospital stay in the EG were shorter (*P* < 0.01, Figure 1).

### SAS and SDS scores between the two groups

**Figure 2:**
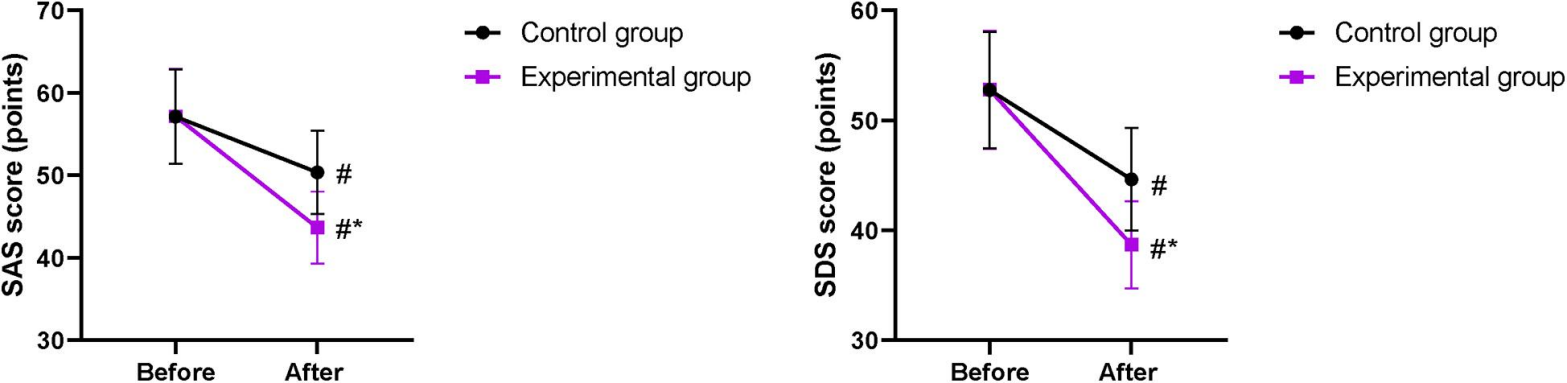
SAS and SDS scores between the two groups. ^#^*P* < 0.05, compared with before nursing. *P < 0.05, compared with the CG.

Prior to intervention, no difference was discovered in SAS and SDS scores between the two groups (*P* > 0.05). After intervention, SAS and SDS scores declined in both groups, and those in the EG were lower when compared with those in the CG (*P* < 0.05, Figure 2).

### Incidence of complications between the two groups

Compared with the CG, the incidence of complications in the EG was lower (*P* < 0.05, Table 1).

**Table 1:** Incidence of complications between the two groups.

Groups	Cases	Biliary leakage	Shock	Intraperitoneal hemorrhage	Infection	Total incidence rate
Control group	50	1	2	3	3	9 (18.00%)
Experimental group	50	0	0	1	1	2 (4.00%)
*χ* ^2^						5.005
*P*						0.025

### Changes in ALT, TBIL, and DBIL levels between the two groups

**Figure 3:**
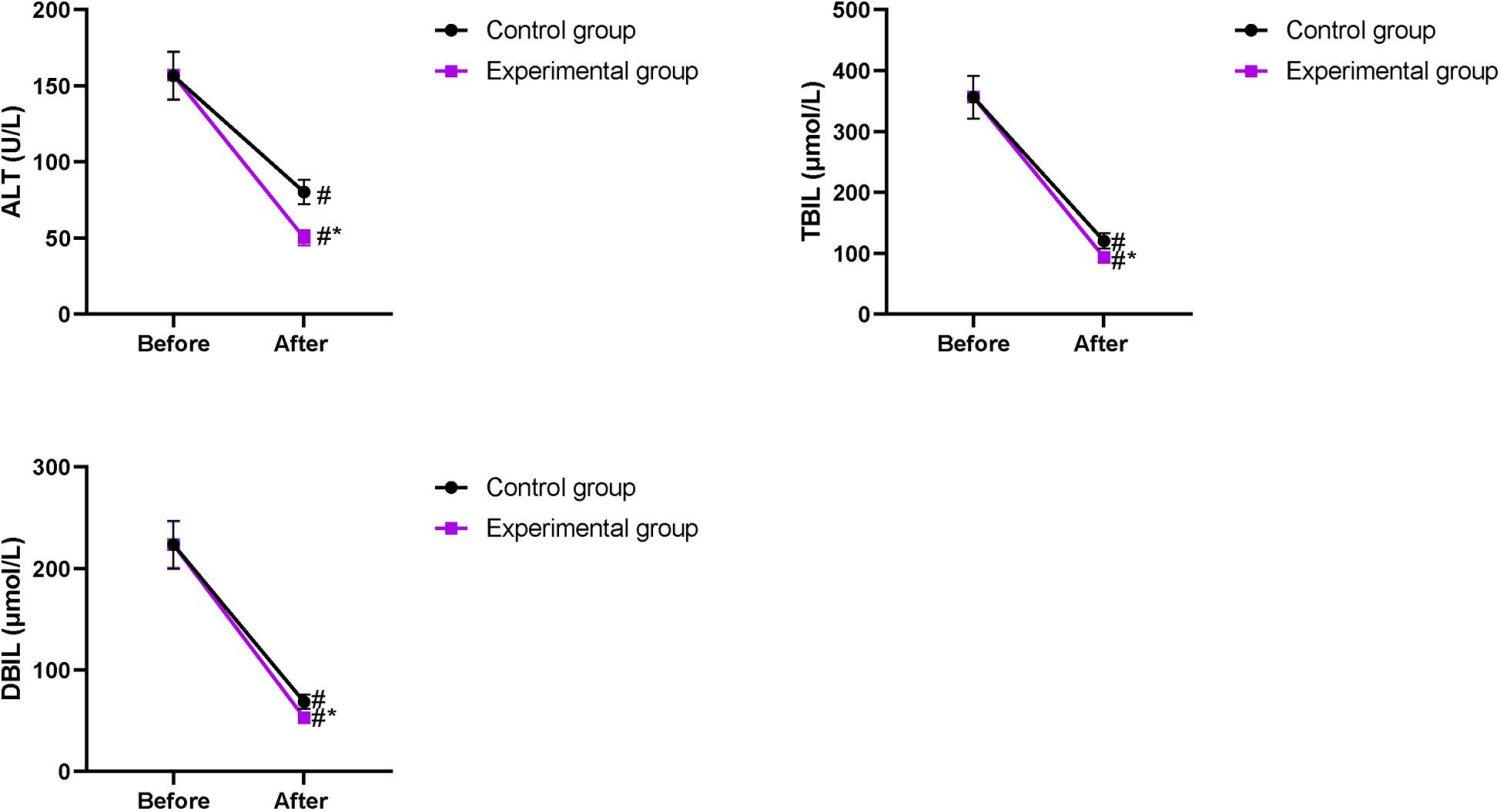
Changes in ALT, TBIL, and DBIL levels between the two groups. ^#^*P* < 0.05, compared with before intervention. *P < 0.05, compared with the CG.

Prior to intervention, no difference was discovered in ALT, TBIL, and DBIL levels between the two groups (*P* > 0.05). After intervention, ALT, TBIL, and DBIL levels declined in both groups, and those in the EG were lower compared with those in the CG (*P* < 0.05, Figure 3).

### Nutritional status between the two groups

**Figure 4:**
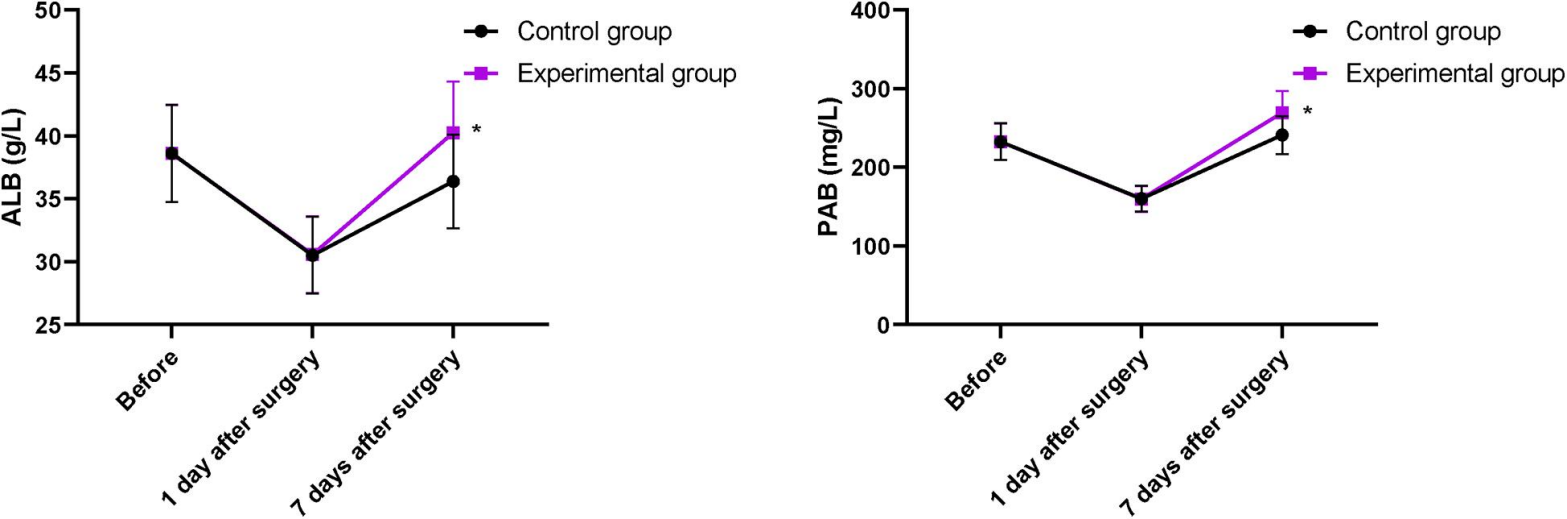
Nutritional status between the two groups. *P < 0.05.

Serum ALB and PAB levels in both groups on the first day after operation significantly decreased compared with those before operation, but the difference between the two groups was not statistically significant (*P* > 0.05). Serum ALB and PAB levels increased in both groups on the 7th day of treatment, and those in the EG were higher compared with those in the CG (*P* < 0.05, Figure 4).

### Immune function in the two groups

**Figure 5:**
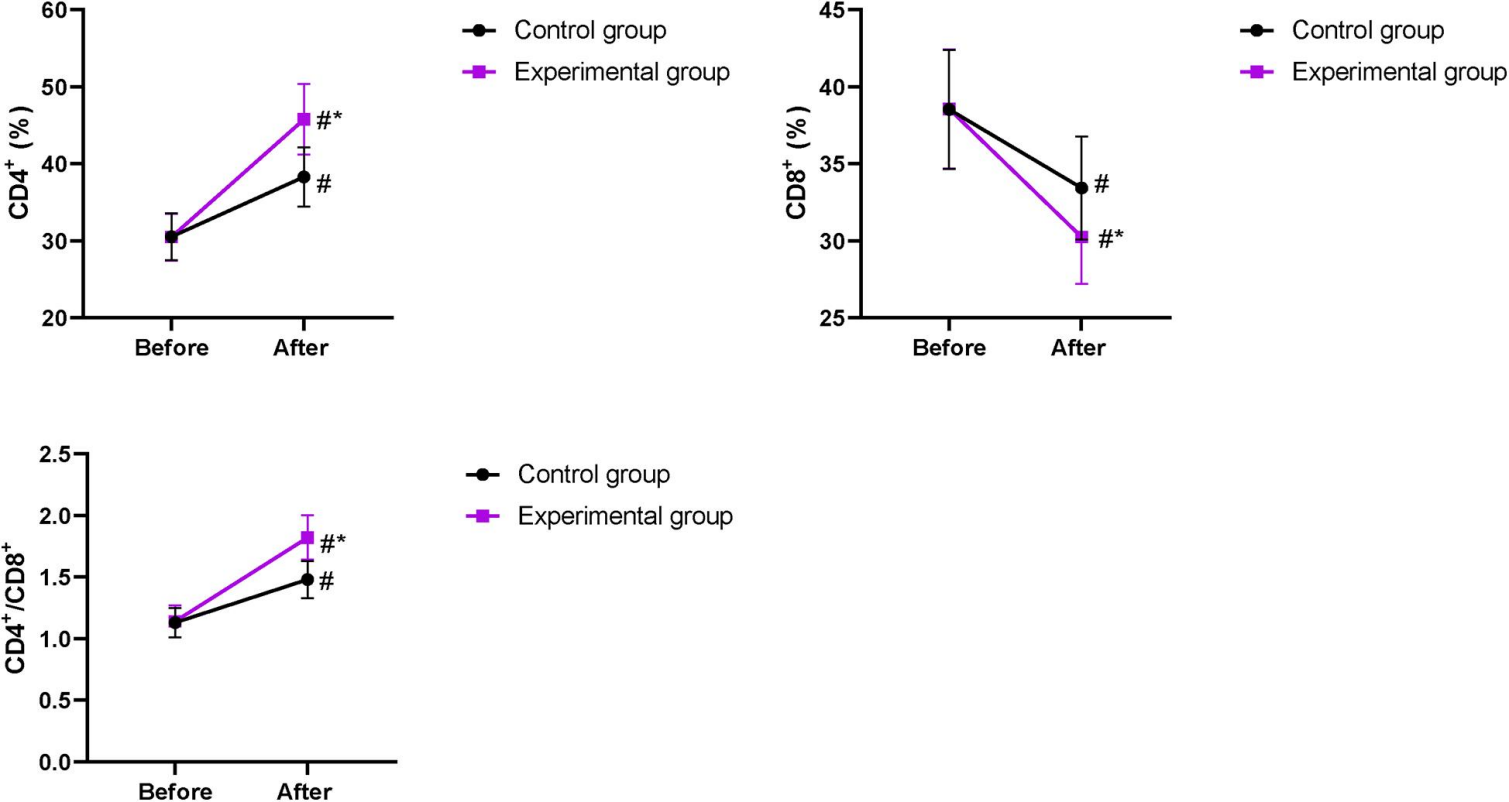
Immune function in both groups. ^#^*P* < 0.05, compared with before intervention. *P < 0.05, compared with the CG.

Prior to intervention, no difference was discovered in CD8^+^, CD4^+^, and CD4^+^/CD8^+^ levels between the two groups (*P* > 0.05). After intervention, CD8^+^ levels declined, while CD4^+^ and CD4^+^/CD8^+^ levels elevated in both groups, and the improvements in CD8^+^, CD4^+^, and CD4^+^/CD8^+^ levels were more pronounced in the EG compared with those in the CG (*P* < 0.05, Figure 5).

### Quality of life between the two groups

**Figure 6:**
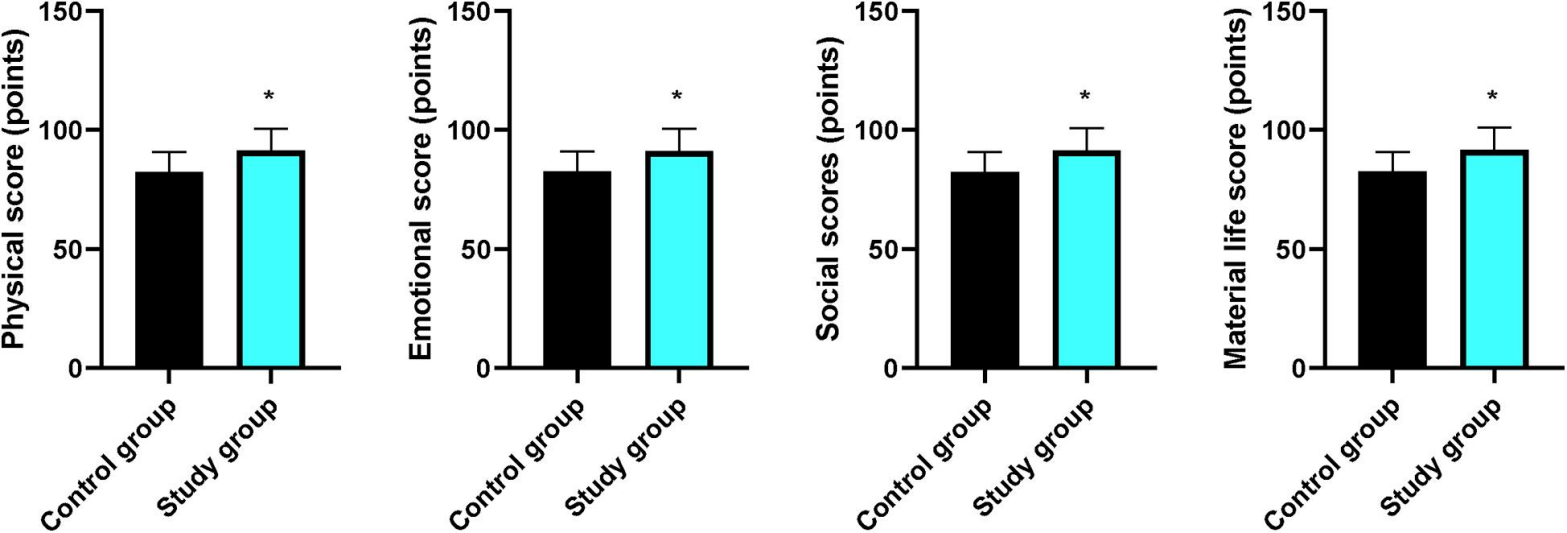
Quality of life between the two groups. *P < 0.05.

Compared with the CG, the scores for physical, emotional, social, and material life in the EG were higher (*P* < 0.05, Figure 6).

### Nursing satisfaction between the two groups

Compared with the CG, the nursing satisfaction of patients in the EG was better (*P* < 0.05, Table 2).

**Table 2:** Nursing satisfaction in both groups.

Groups	Cases	Satisfied	Basically satisfied	Dissatisfied	Total satisfaction rate
Control group	50	25	15	10	40 (80.00%)
Experimental group	50	30	19	1	49 (98.00%)
*χ* ^2^					8.274
*P*					0.004

## Discussion

The main harm of malignant obstructive jaundice lies in the functional impairment of multiple organs throughout the body as the disease progresses, especially the liver (15). Without timely and effective treatment, further deterioration of liver function can reduce immunoglobulin secretion and limit lymphocyte differentiation and proliferation, leading to a state of immunosuppression. This will, in turn, aggravate liver damage, resulting in a vicious cycle and creating great damage to patients’ overall health (16).

At present, PTCD remains the most effective treatment method for malignant obstructive jaundice with small trauma, convenient operation, and obvious effect, which can complete biliary drainage to a certain extent and reduce and improve jaundice (17). However, this process still cannot avoid invasion, and long-term catheterization has high intervention requirements for preventing catheter blockage and leakage (18). Therefore, during treatment, nursing staff must maintain a strong sense of responsibility, exercise keen observation, closely monitor the patient’s condition, and implement effective interventions to prevent complications. These measures are crucial for improving patient prognosis.

Based on the development of the Internet and the popularity of mobile phones, online platform learning has become a new way of learning for people (19). “Internet + nursing service” can understand patients' physical status in real time through WeChat, apps, and other tools; use big data to analyze patients’ diagnosis, treatment, and rehabilitation information; sum up the best nursing methods; and solve patients' sudden adverse events in a timely manner (20). The platform education department pushes PTCD postoperative rehabilitation nursing methods and precautions through videos and documents every day and holds expert lectures to answer the doubts of patients and their families and to improve the cognition level of patients and their families on PTCD knowledge and postoperative precautions. In addition, the “Internet + nursing service” is equipped with a psychologist for patients. Through the mixed communication methods of online and offline, psychologists can timely understand the psychological demands of patients, provide psychological counseling to patients, relieve patients' worries and difficulties, and enhance the confidence of patients and their families to fight against the disease.

The positive outcomes associated with our “Internet + nursing service” model find resonance in the global expansion of digital health, or mHealth, initiatives. While the term “Internet +” is prominent in China, the concept of using technology to provide continuous care outside the hospital is a worldwide trend. For instance, studies from Europe and North America have demonstrated that telehealth follow-up for patients with complex chronic conditions or postsurgical care can improve patient satisfaction and quality of life, similar to our findings (21). However, the specific integration of a multidisciplinary team (including surgeons, dietitians, and psychologists) via a centralized platform, as implemented in our study, represents a model that could be adapted and tested in other healthcare systems. A review by Noah et al. (22) highlighted that the success of such interventions is highly dependent on cultural context and healthcare infrastructure, suggesting that while our results are promising, their transferability requires further investigation in international settings.

After the treatment of malignant obstructive jaundice, it is particularly important to give timely nutritional treatment (23). At present, nutrition support treatment is mainly divided into enteral and parenteral nutrition support, and the two treatment schemes give nutrients in different ways; enteral nutrition support is through the gastrointestinal tract, while parenteral nutrition support is through the intravenous route (24). Although total parenteral nutrition support is the main treatment plan at present, the advantage is that it can significantly improve the prognosis of patients, but the disadvantage is that long-term application will cause a series of complications, such as catheter infection, intestinal mucosal damage and atrophy, and metabolic disorders (25). Early enteral nutrition support provides relevant nutrients directly to the intestinal mucosa, reduces the secretion of pancreatic fluid and the release of inflammatory mediators, and can improve the immune function of patients to some extent (26). Moreover, another advantage of early enteral nutrition support is to effectively maintain the gastrointestinal mucosal barrier function of patients, ensure the increase of liver blood flow, improve the normal metabolic function and repair ability of patients, and improve immune function (27). The guidelines from the European Society for Clinical Nutrition and Metabolism (ESPEN) strongly recommend the implementation of early enteral nutrition in surgical patients where possible, as it helps maintain gut barrier function and modulate the systemic inflammatory response (28). A meta-analysis focusing on patients undergoing pancreaticoduodenectomy concluded that early enteral nutrition was associated with a significant reduction in hospital stays compared with parenteral nutrition (29).

In our study, the results displayed that in contrast to the CG, the time of first exhaust, the time of first defecation, and the length of hospital stay in the EG were shorter, and the incidence of complications in the EG was lower suggesting that early enteral and parenteral nutrition support combined with “Internet + nursing service” could promote the postoperative recovery and reduce the incidence of complications in patients with malignant obstructive jaundice after PTCD, which was consistent with previous reports (30, 31).

Our study indicated that after intervention, ALT, TBIL, and DBIL levels in the EG were lower compared with those in the CG; serum ALB and PAB levels in the EG were higher compared with those in the CG on the 7th day of treatment; and the improvements in CD8^+^, CD4^+^, and CD4^+^/CD8^+^ levels in the EG were more pronounce compared with those in the CG, suggesting that early enteral and parenteral nutrition support combined with “Internet + nursing service” was beneficial to the recovery of liver function and promoted the nutritional status and immune function of patients with malignant obstructive jaundice after PTCD. Consistently, Zhu et al. (32) have indicated that parenteral nutrition supplementation combined with enteral nutrition support can greatly improve the nutritional state and liver function of patients undergoing pancreaticoduodenectomy.

Moreover, our study demonstrated that after intervention, SAS and SDS scores in the EG were lower compared with those in the CG; the scores for physical, emotional, social, and material life in the EG were higher compared with those in the CG; and the nursing satisfaction of patients in the EG was better compared with that in the CG, implying that early enteral and parenteral nutrition support combined with “Internet + nursing service” could relieve the negative emotions and promote the quality of life and the nursing satisfaction of patients with malignant obstructive jaundice after PTCD, which was in line with previous study (33).

This study has several limitations that should be considered when interpreting the results. Firstly, this was a single-center study with a relatively small sample size (*n* = 100). The single-center design may limit the heterogeneity of the patient population and standardization of care, potentially affecting the generalizability of our findings to other settings with different healthcare practices and patient demographics. The sample size, while sufficient to detect statistically significant differences in our primary outcomes, may be underpowered to detect rarer complications or subtler effects. Therefore, the results should be interpreted with caution, and their external validity needs to be verified in future studies. Secondly, due to the nature of the complex interventions involving different nursing models and nutritional support routes, blinding of the participants and healthcare providers was not feasible, which might introduce potential performance bias. Future research should involve larger, multicenter, randomized controlled trials to confirm our conclusions and enhance the generalizability of the findings.

## Conclusion

Our study indicates that early enteral and parenteral nutrition support combined with “Internet + nursing service” can promote the quality of life and the nutritional status and immune function and reduce the complications in patients with malignant obstructive jaundice after PTCD.

## Data Availability

The raw data supporting the conclusions of this article will be made available by the authors, without undue reservation.
